# Supplementary material for: Handheld dynamometry: Validity and reliability of measuring hip joint rate of torque development and peak torque
Source: PLoS One. 2024 Aug 16;19(8):e0308956. doi: 10.1371/journal.pone.0308956 (PMC11329127; doi:10.1371/journal.pone.0308956)

**Handheld dynamometry: validity and reliability of measuring hip joint rate of torque development and peak torque.**

S1 Appendix: Bland-Altman plots to show agreement of HHD with IKD measuring RTD in hip movements for group results.


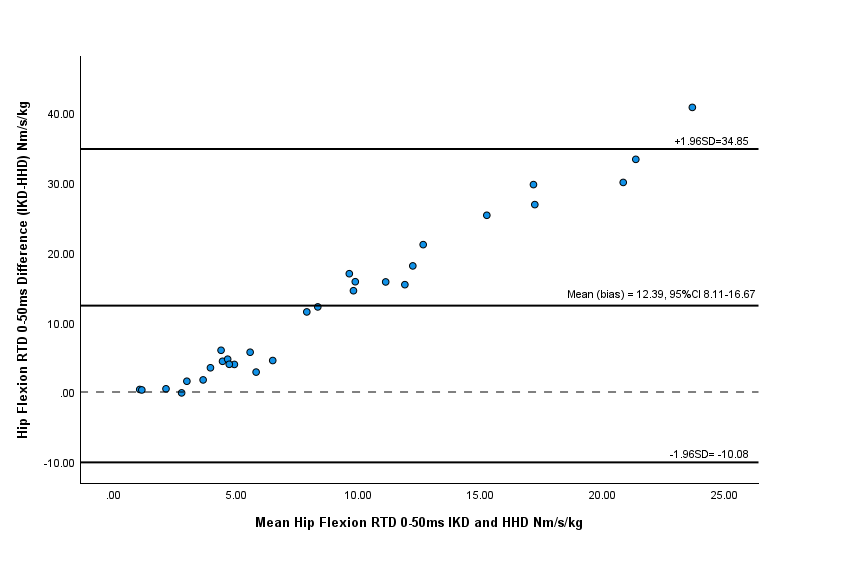
**RTD 0-50ms**


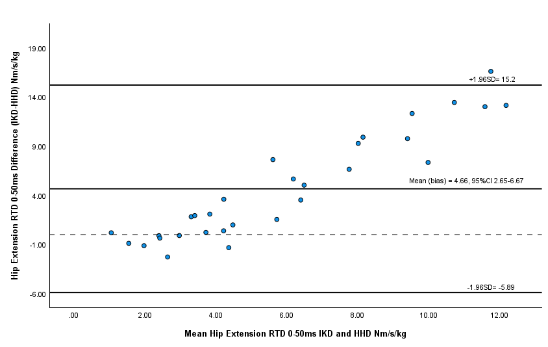


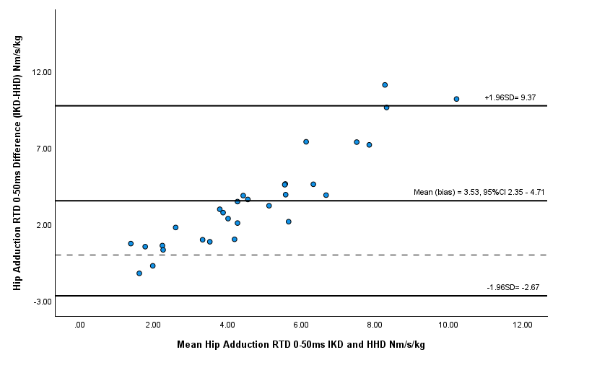

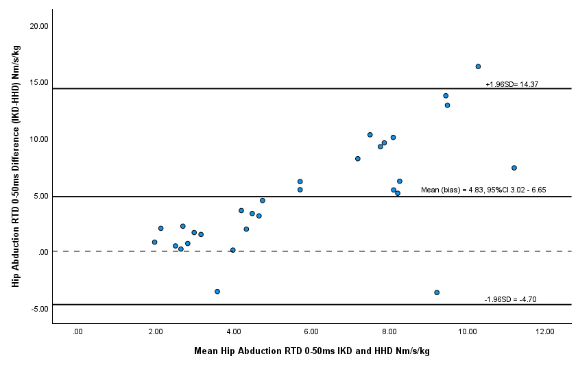


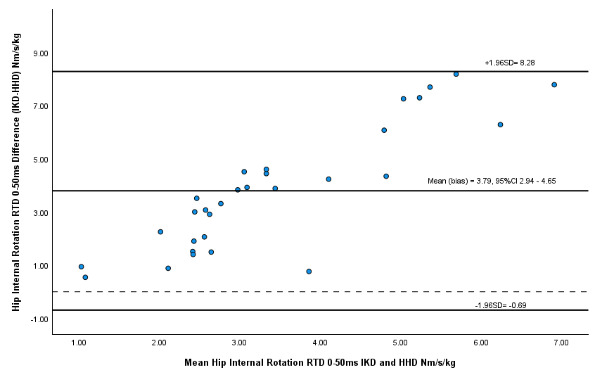


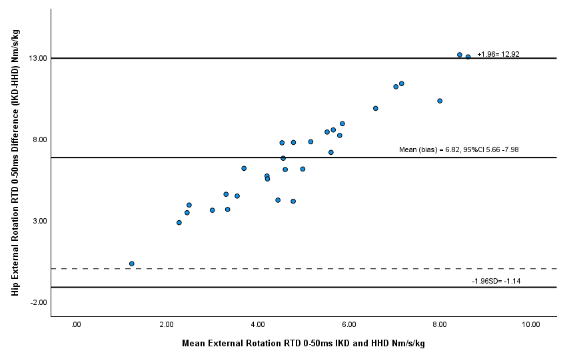


**RTD 0-100ms**


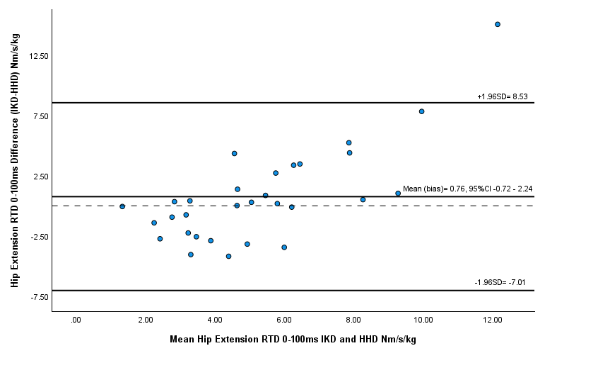

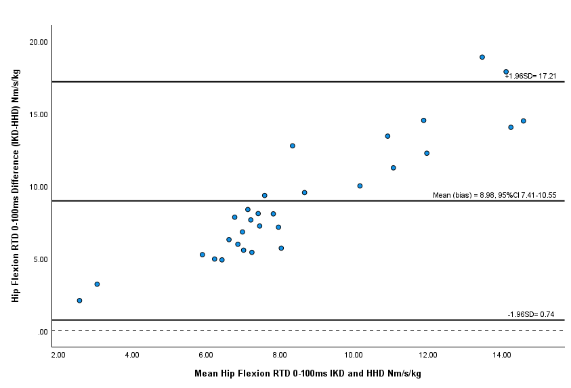


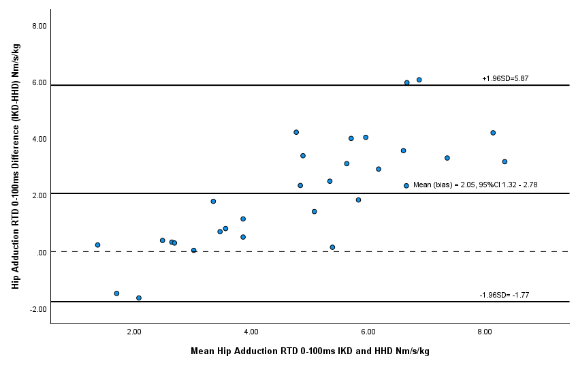

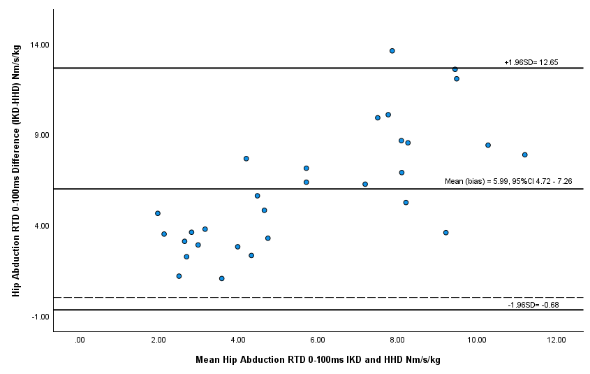


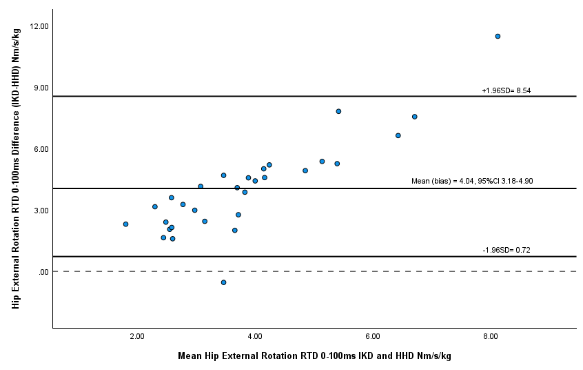

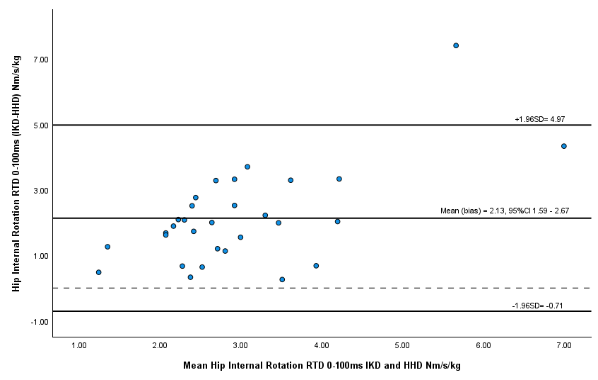


**RTD 0-150ms**


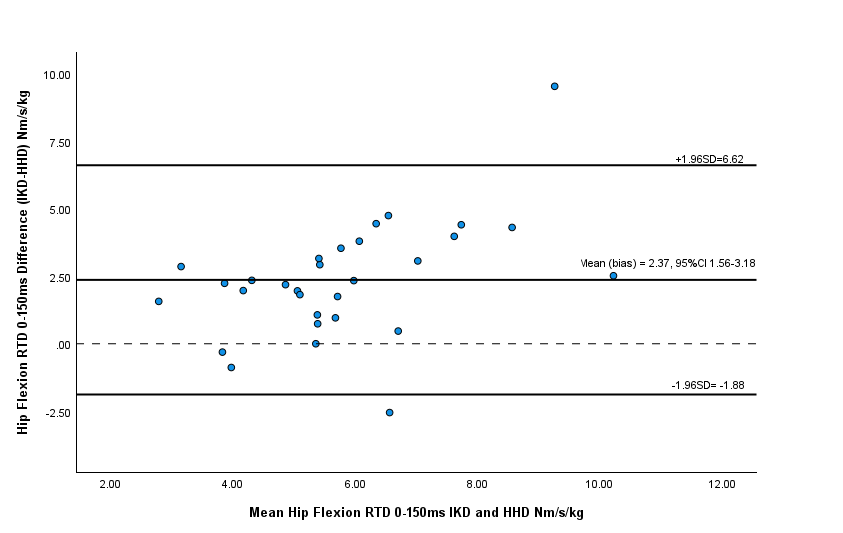

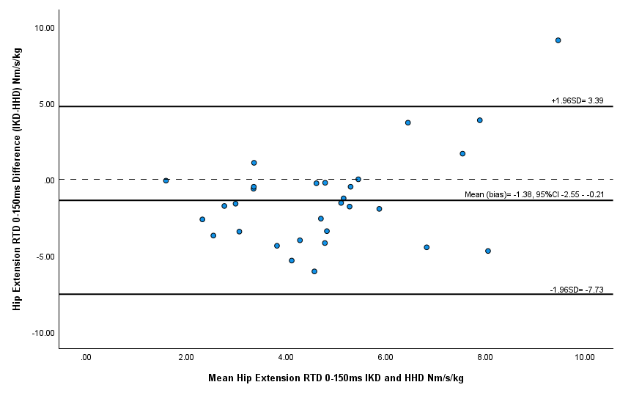


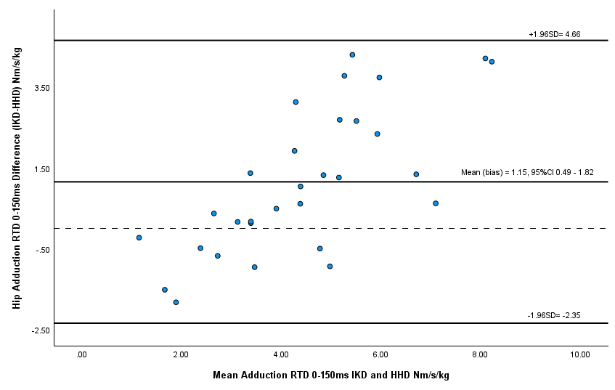

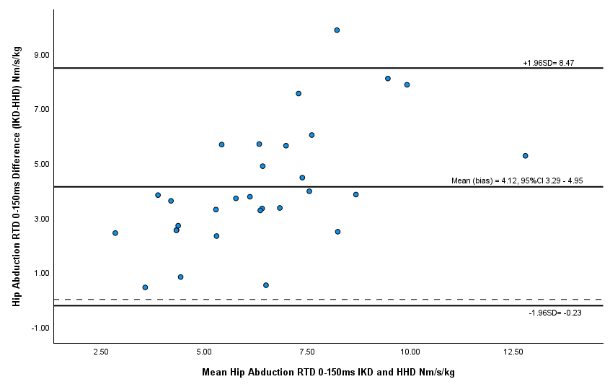


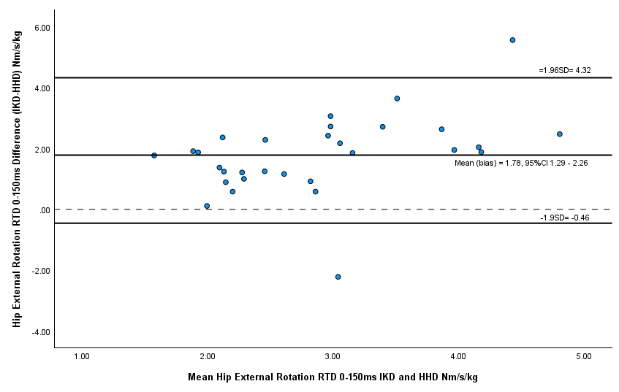

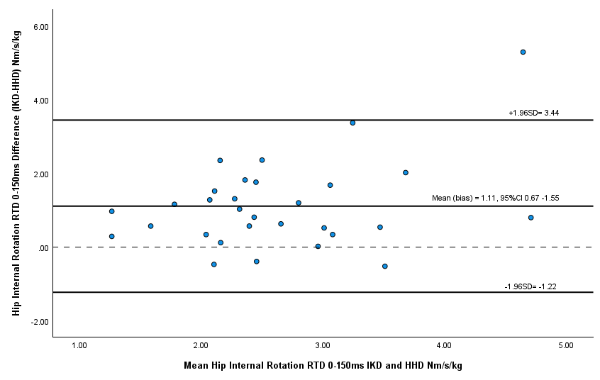


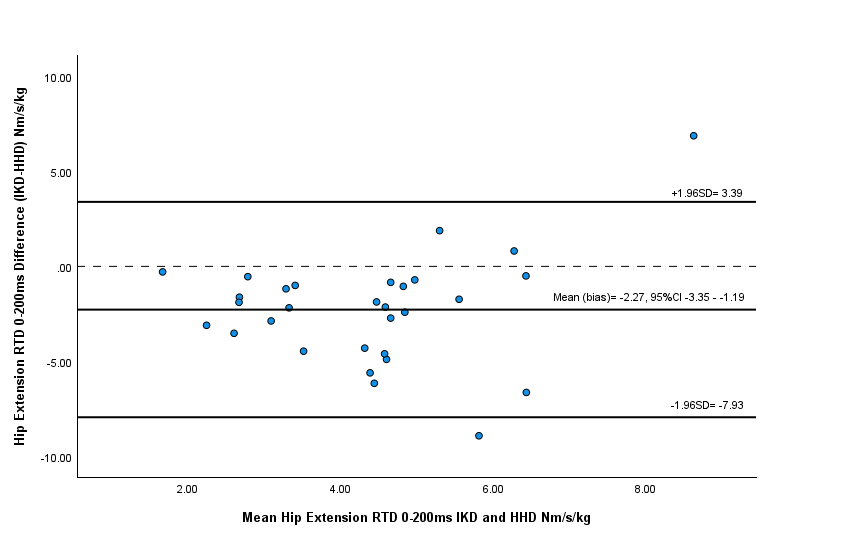

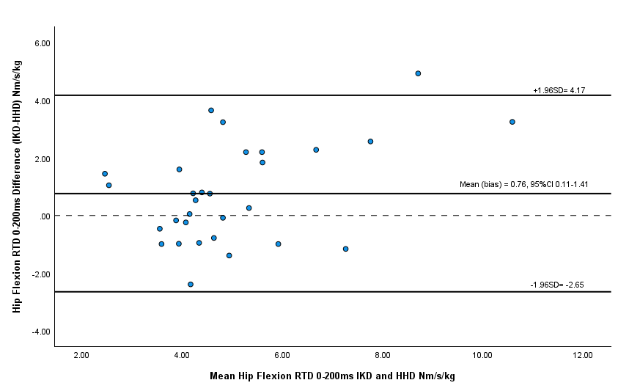
**RTD 0-200ms**


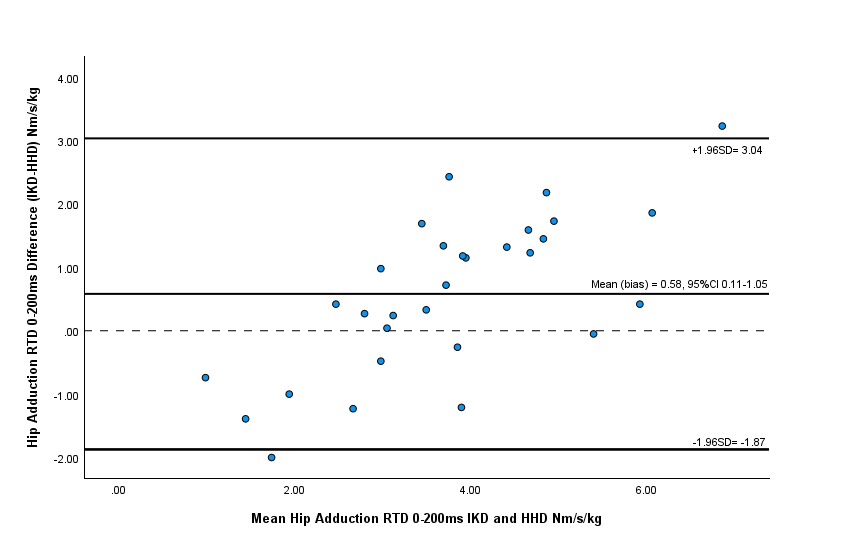

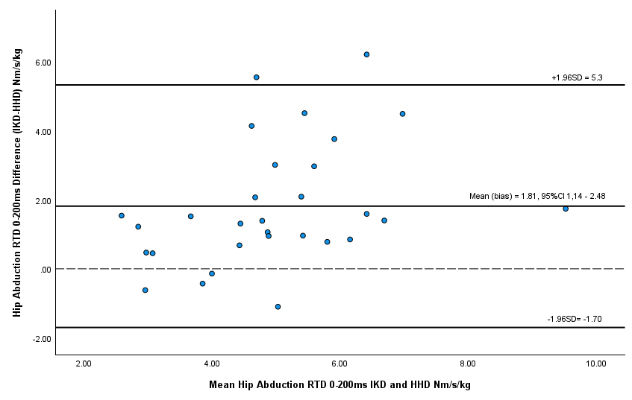


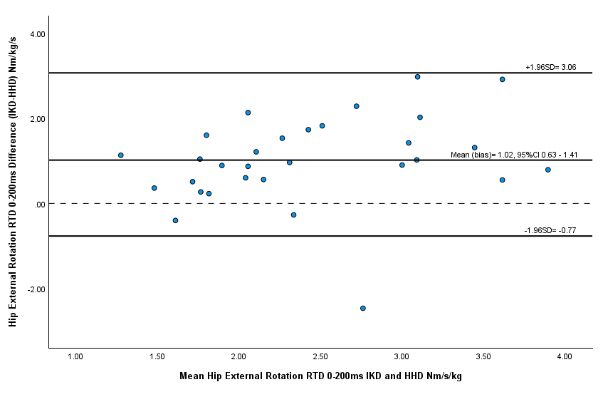

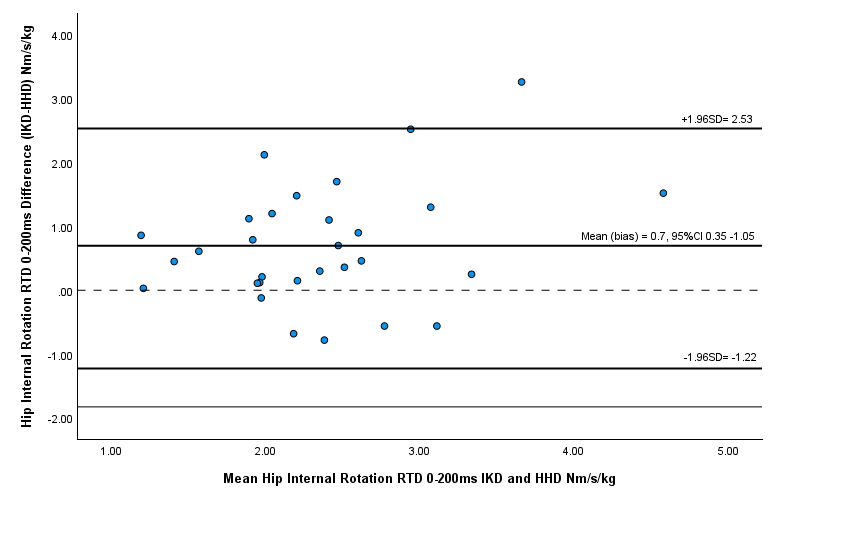

Supplement: S1 File — (DOCX) [file pone.0308956.s002.docx]
