# Supplementary material for: Handheld dynamometry: Validity and reliability of measuring hip joint rate of torque development and peak torque
Source: PLoS One. 2024 Aug 16;19(8):e0308956. doi: 10.1371/journal.pone.0308956 (PMC11329127; doi:10.1371/journal.pone.0308956)

**Handheld dynamometry: validity and reliability of measuring hip joint rate of torque development and peak torque.**

S2 Appendix: Bland-Altman plots to show agreement of HHD with IKD measuring peak torque in hip movements for group results.


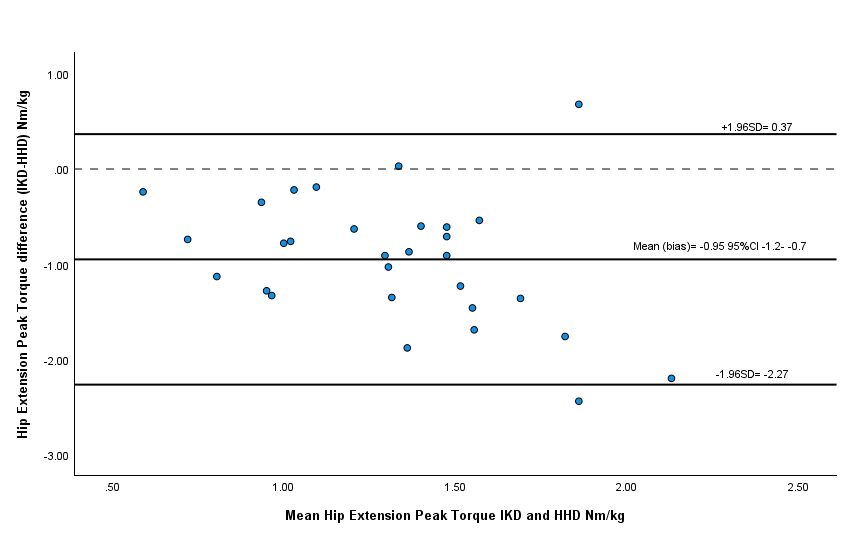

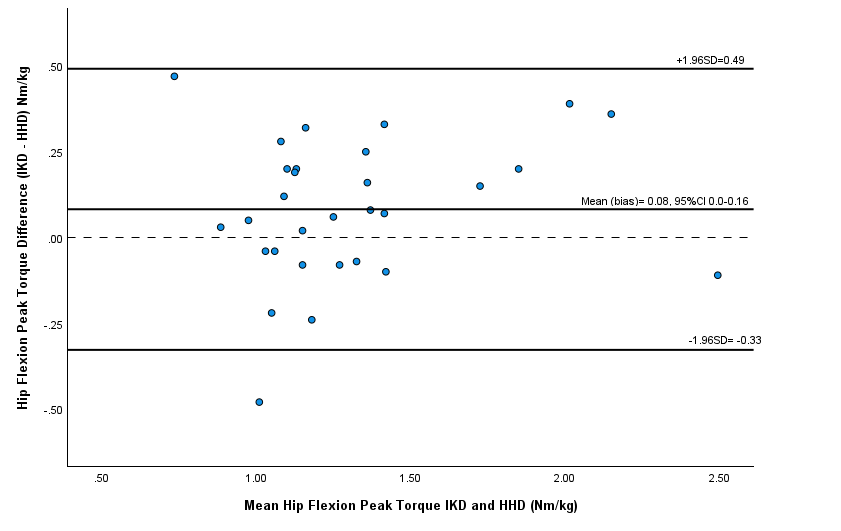


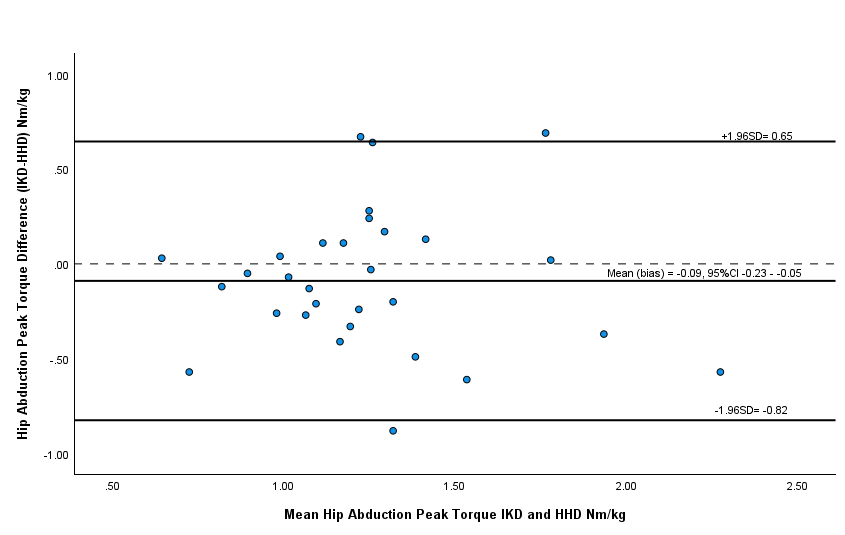

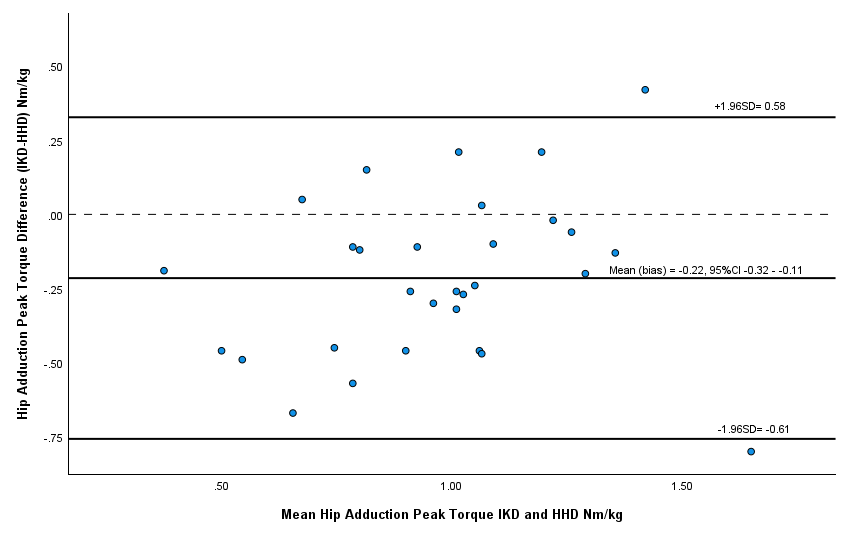


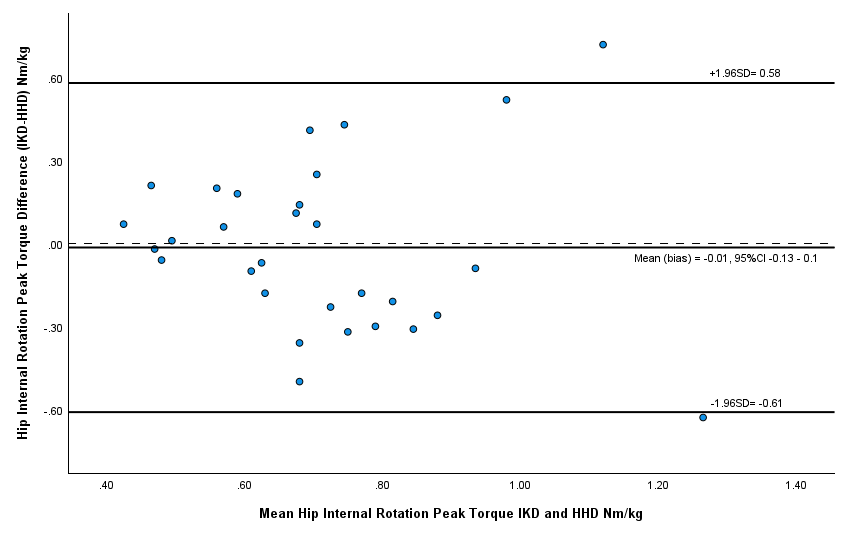

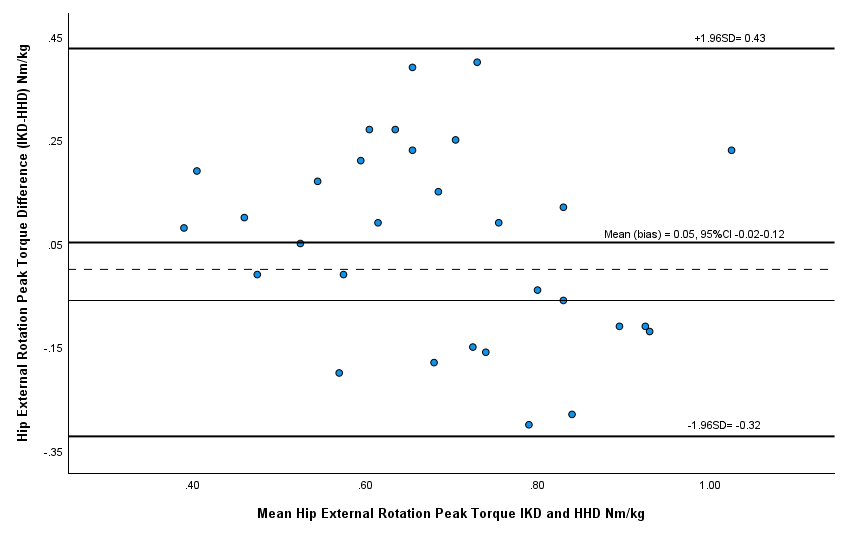

Supplement: S2 File — (DOCX) [file pone.0308956.s003.docx]
