# Supplementary material for: Handheld dynamometry: Validity and reliability of measuring hip joint rate of torque development and peak torque
Source: PLoS One. 2024 Aug 16;19(8):e0308956. doi: 10.1371/journal.pone.0308956 (PMC11329127; doi:10.1371/journal.pone.0308956)
Supplement: Attachment — Submitted filename: Response to Reviewers PONE-D-24-01588R1.docx [file pone.0308956.s004.docx]

**Comments from the editors and reviewers:**

We would like to thank the Editor and the Reviewers for their comments, observations and suggestions. Please find our responses below where we hope to have addressed all these elements. Where these generated changes in the manuscript, the correct location is indicated and is highlighted within the manuscript’s text. An unmarked version of the revised paper without tracked changes has also been submitted.

**1.Please include a version (it can even be a shortened one) of your reply to R2 re the 'the clinical importance of any potential bias' in the manuscript, so the reader is explicitly informed about the difficulty in determining that.**

‘Thank you for your comment. We have inserted a paragraph into the discussion section to address this. The manuscript ‘Discussion’ section reads as follows.

The agreement analysis further highlights a statistically significant systematic difference between the two devices across all hip movements, indicating the HHD consistently yielded lower RTD values compared to the IKD. Consequently, while the results from the two devices are not interchangeable, in clinical practice where only one device is employed for assessment, achieving agreement with another device may not be as crucial.

Due to the limited literature specifically examining RTD measurements between dynamometers, the clinical consequences of bias in a particular direction were not predefined. Instead, trends in agreement were identified, noting that later-phase RTD measurements exhibited lower bias and narrower confidence intervals. While establishing clinically meaningful thresholds for bias is important, valuable insights into the agreement between measurements from HHDs and IKDs are still provided by the findings from this present study. Now that it is understood the HHD consistently measured lower than IKD, these findings can help inform clinical decision-making and guide future research on setting acceptable levels of bias. Further research is needed to help define acceptable limits of agreement in RTD measurements between dynamometers.’

**2.Additionally, please standardise reporting as either 'CI' or '95%CI' (preferable).**

This has been updated to 95%CI throughout the manuscript, including on the Bland and Altman plots. Changes have been highlighted.
